# Antibody Targeting of Eph Receptors in Cancer

**DOI:** 10.3390/ph13050088

**Published:** 2020-05-08

**Authors:** Peter W. Janes, Mary E. Vail, Hui K. Gan, Andrew M. Scott

**Affiliations:** Olivia Newton-John Cancer Institute and La Trobe University School of Cancer Medicine, Victoria 3084, Australia; mary.vail@onjcri.org.au (M.E.V.); hui.gan@onjcri.org.au (H.K.G.); andrew.scott@onjcri.org.au (A.M.S.)

**Keywords:** Eph receptor, therapeutic antibodies, cancer

## Abstract

The Eph subfamily of receptor tyrosine kinases mediate cell-cell communication controlling cell and tissue patterning during development. While generally less active in adult tissues, they often re-emerge in cancers, particularly on undifferentiated or progenitor cells in tumors and the tumor microenvironment, associated with tumor initiation, angiogenesis and metastasis. Eph receptors are thus attractive therapeutic targets, and monoclonal antibodies have been commonly developed and tested for anti-cancer activity in preclinical models, and in some cases in the clinic. This review summarizes 20 years of research on various antibody-based approaches to target Eph receptors in tumors and the tumor microenvironment, including their mode of action, tumor specificity, and efficacy in pre-clinical and clinical testing.

## 1. Introduction

Eph receptors (first isolated from an Erythropoietin-Producing Hepatocellular carcinoma) are receptor tyrosine kinases (RTKs) that mediate cell-cell interactions with their cell-bound ephrin ligands, controlling adhesion and migration, and influencing proliferation and cell fate. Ephs make up the largest family of RTKs, with 14 members classified into two subtypes, A and B, distinguished by sequence similarity, and their preferential binding to A- and B-type ephrins, respectively. The six A-type ephrins are GPI (glycophosphatidylinositol)-linked, whereas the three B-type ephrins are transmembrane proteins [1,2]. Eph-ephrin interaction results in bi-directional signaling in contacting cells, resulting in either cell-cell adhesion (associated with migration and invasion), or cell-cell repulsion (resulting in cell segregation), dependent on the relative expression and affinity of ligand/receptor pairs, and receptor tyrosine kinase activity. Thus, ligand-binding in the context of low expression (or affinity) of receptors and ligands tends to promote adhesion, whereas binding between highly expressed, high affinity partners enables extensive receptor clustering, autophosphorylation and kinase activity, resulting in cytoskeletal reorganization and cell retraction. This can be further modulated by tyrosine phosphatase activity [3] and cross-talk with other signaling pathways [4]. Ephs and ephrins are expressed widely throughout development, regulating tissue and organ boundary formation, and patterning of the neural and vascular systems. They are less expressed in adult tissues, but reappear in a variety of cancer types, both in tumor cells and the tumor microenvironment (TME), where they can mediate similar processes such as tumor neo-angiogenesis, invasion and metastasis [1,2]. Ephs are often expressed on less differentiated, progenitor or ‘stem’-like tumor cell populations, which are associated with tumor initiation, metastasis and resistance to therapy [5]. Interestingly, over-expressed Ephs often appear to have low kinase activity in tumors, and mutations thought to inhibit activity have been reported in various cancer types, suggesting a kinase-independent oncogenic role of Eph receptors [4]. Furthermore, in some contexts, loss of Eph expression can also promote certain stages of cancer progression, reflecting the complex nature of Eph signaling in cancer [4,6]. Despite this complexity, Eph receptors remain promising targets for therapeutic intervention in cancer. Moreover, their location on the cell surface make them accessible to targeting with a wide range of agents, including larger molecules unable to passively cross cell membranes.

**Figure 1:**
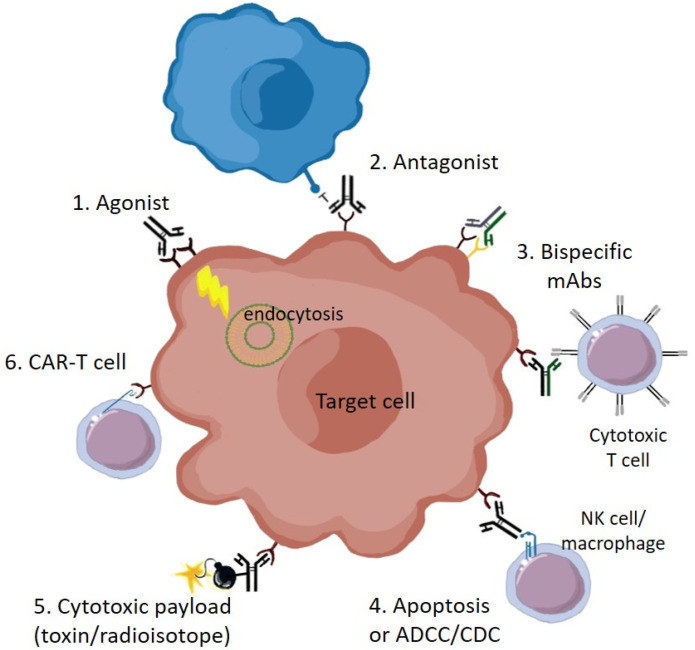
Therapeutic mechanisms of anti-Eph receptor antibodies: (1) Agonist mAbs can promote receptor clustering, tyrosine phosphorylation, signaling (with downstream effects on cell behavior such as cytoskeletal rearrangement), internalization and degradation; (2) Antagonist mAbs inhibit Eph receptor function, such as by blocking binding to ligand on adjacent cells (blue); (3) Bispecific antibodies, which concurrently bind distinct receptor types on the same cell, or on different cell types, such as to recruit T cells; (4) Apoptosis, or cell death, either directly due to signaling effects, or by immune-mediated ADCC (antibody-dependent cell-mediated cytotoxicity), or CDC (complement-dependent cytotoxicity); (5) Conjugation of cytotoxic drugs or radioactive isotopes; (6) Use of mAb antigen-binding domains for targeting of CAR-T cells. The target Eph-expressing cell could be either a transformed tumor cell or a non-transformed cell in the tumor microenvironment, contributing to the stroma, vasculature or immune regulation.

Antibodies have become favored as therapeutics, due to their high specificity, affinity, and stability, with relatively long half-life in the body compared to small molecule inhibitors [7]. Antibodies that bind to cell surface proteins in general can have multiple mechanisms of action, and this is certainly the case for antibodies against Eph receptors (Figure 1). These include: (1) receptor activation, or agonism, by causing receptor clustering and signaling—this can also lead to receptor endocytosis and degradation, and subsequently reduced receptor levels; (2) inhibition, or antagonism, such as through blocking of ligand binding; (3) cytotoxicity, through direct induction of apoptosis, or by immune attack via antibody-dependent cell-mediated cytotoxicity (ADCC), or complement-dependent cytotoxicity (CDC); or, (4) delivery of a cytotoxic payload, such as a radioactive isotope, or a conjugated drug (antibody-drug conjugate, ADC) or drug-containing nanoparticle [8,9]. Moreover, bispecific antibodies can be engineered to target more than one antigen, even on distinct cell types. Antibody binding regions can furthermore be incorporated into engineered CAR (Chimeric Antigen Receptor)-T cells, to directly target cytotoxic T cells to tumors expressing antigen [10]. For these reasons, over 20 monoclonal antibodies (mAbs) are now approved for cancer treatment, with several targeting RTKs, and a similar number against immune modulatory targets [8].

It is therefore not surprising that antibodies have been the most preferred method for development of Eph-targeted therapeutics. A range of antibodies targeting Eph receptors have been investigated pre-clinically, some of which have been tested in clinical trials (Table 1). These have had varying success, which likely reflects the range of possible mechanisms of antibody action, as well as the degree of tumor specific target expression and the complexities of Eph-ephrin signaling, indicated above [4]. While the complex nature of Eph-ephrin actions in development and cancer have been extensively reviewed elsewhere [1,2,4,5,11], this review will focus on effects of antibodies targeting the Eph-ephrin system in the context of cancer, highlighting the promise and pitfalls evident from nearly two decades of preclinical and clinical research.

## 2. EphA2

EphA2 has been long recognized as a potential therapeutic target, being overexpressed in a range of cancer types, including both mesenchymal tumors such as melanoma and glioma, and epithelial tumors including prostate, breast, ovarian, lung, colon, esophageal, gastric, cervical, and bladder cancers [12]. This reflects its expression in both mesenchymal and epithelial tissues during development [2]. EphA2 has been reported to be associated with cancer stem-cell qualities [5], and to promote tumor neo-angiogenesis [13,14], and metastasis, including cross-talk with erbB receptor signaling to control initiation and spread of erbB2-dependent mammary tumors in mice [15]. Like other Ephs, EphA2 has both kinase dependent and independent signaling functions [16,17,18]. Cell migration and invasion is typically reduced by ligand stimulation, but supported by ligand-independent signaling mediated by the overexpressed receptor [19], underlining the importance of careful assessment of agonistic versus antagonistic targeting.

Initial studies identified anti-EphA2 mAbs with preferential binding to tumor cells that inhibited transformed or metastatic cell behavior in vitro [20,21], and inhibited tumor growth in mice, with anti-vascular effects [22]. These were agonistic antibodies, causing receptor phosphorylation and downregulation, consistent with Eph agonism being tumor-inhibitory. However, it should be noted that not all agonistic EphA2 antibodies have proved effective in vivo [23]. Also, in another study, both an agonist antibody (IgG25) and a ligand blocking antibody (IgG28) were found to have tumor inhibitory effects in a pancreatic xenograft model, the former due to receptor downregulation, the latter due to vascular disruption [24], highlighting the complexity of potential mAb-induced effects.

EphA2 mAbs can also promote anti-tumor immune responses. Agonist EphA2 mAbs caused proteasome-dependent degradation of the receptor, resulting in MHC class I presentation of EphA2 peptides on tumor cells and increased recognition by EphA2-specific CD8+ T cells in vitro. While antibody alone was not sufficient to inhibit tumor growth, adoptive transfer of anti-EphA2 CD8+ T cells led to tumor eradication [25]. An inhibitor of the chaperone protein Hsp90, which stabilizes RTKs including EphA2, also promoted EphA2 degradation and recognition by EphA2-specific CD8+ T cells [26].

Antibody-dependent cell-mediated cytotoxicity (ADCC) can further promote anti-tumor activity of EphA2 mAbs. This is an immune mechanism where Fc receptor-bearing effector cells recognize and kill antibody-bound target cells, which is important for various therapeutic antibodies such as rituximab (anti-CD20 mAb) and trastuzumab (anti-erbB2 mAb) [7]. A role for ADCC was shown by studies using a humanized form of the B233 antibody (3F2-3M) with a modified Fc region to promote recognition by mouse and human Fcγ receptors [27]. 3F2-3M treatment elicited natural killer (NK) cell-dependent ADCC, as shown by enhanced tumor cell killing in mice with functional NK cells compared to those with compromised NK cell function [27]. More recently, the humanized EphA2 mAb DS-8895a, modified to be afucosylated to enhance ADCC, was effective in inhibiting growth of breast and gastric xenograft models [28].

## 3. EphA3

EphA3 has also been a target for therapeutic antibodies. EphA3 is expressed in mesenchymal tissues during development, is generally absent or low expressed in homeostatic adult tissues, but reappears in a range of cancer types [41,60]. It was originally identified as a tumor antigen using an antibody (IIIA4) raised against a lymphoblastic leukemia cell line [61]. It was independently identified on tumor cells from a melanoma patient by virtue of an EphA3-reactive T cell immune response [62]. EphA3 was subsequently found to be widely expressed also in myeloid malignancies [63,64], and in tumors of mesenchymal origin, such as melanoma, sarcoma and glioblastoma, where it is particularly associated with a more mesenchymal phenotype, and progenitor or stem cell-like tumor cells [49]. EphA3 is also expressed in a wide range of epithelial tumors, often as part of the tumor microenvironment (TME), particularly in the mesenchymal-derived stromal and vascular tissues supporting the tumor, and in some myeloid-derived immune cell subtypes [41,65]. EphA3+ mesenchymal stromal cells are associated with neo-angiogenesis in regenerating endothelium [66], and in tumors [41], similar to EphA2.

The EphA3 mAb IIIA4, used to identify the receptor, has been investigated as a potential therapeutic agent. It was found to be agonistic, and enhanced ligand-mediated receptor activation [42]. IIIA4 treatment decreased tumor growth and infiltration of secondary tissues in the LK63 pre-B ALL cell line xenograft model [43]. It also inhibited growth of prostate and colon xenografts, particularly targeting EphA3+ stromal cells in the TME, and inhibiting angiogenesis [41]. Similarly, IIIA4 treatment resulted in significant inhibition of tumor growth and angiogenesis in two multiple myeloma-derived mouse xenograft models [44].

## 4. EphBs

Similar to EphA receptors, EphB receptors play critical roles in development, such as in controlling cell-cell adhesion and segregation during tissue boundary development and angiogenesis, exemplified by EphB4 and ephrinB2 interactions [67]. They also re-emerge in various cancer types, with both tumor promoting and suppressive roles reported, sometimes even in the same tumor at different stages. This is exemplified by colon cancer, where EphB2, 3 and 4 are often overexpressed initially, and associated with stem-like self-renewal behavior, but can become lost during progression, promoting invasion and metastasis [68]. As with EphAs, over-expressed EphB receptors in cancers often display low activity, suggesting ligand-independent tumor promoting roles [69].

Of the EphBs, EphB4 has been the most common target for antibodies. Two antibodies against the fibronectin domains in EphB4, mAb47 and mAb131, were found to inhibit tumor growth in a range of EphB4-expressing xenograft models, including prostate, colon, head and neck, and ovarian cancer [54]. Interestingly, these appeared to function differently: mAb131 recognized only human EphB4 on the tumor cells, and caused receptor endocytosis and degradation; whereas mAb47, recognizing also mouse EphB4, severely blocked blood vessel perfusion in tumors, and was also active against tumors in which the tumor cells did not express EphB4, consistent with targeting the TME. Interestingly, both mAbs reduced blood vessel density [54]. More recently, mAb131 was also shown to be effective in acute myeloid leukemia (AML) [70]. Another mAb (C2), targeting the cysteine-rich domain of EphB4, also inhibited the tube-forming behavior of MDA-MB-231 breast tumor cells in vitro (indicative of vasculogenic behavior), and tumor growth of xenografts; however xenografts of PC3 prostate cells were unaffected, thought to be due to their low cell surface receptor expression [55].

## 5. Ephrins

Ephrins have received much less attention than their cognate receptors as targets for therapeutic antibodies. In terms of unconjugated antibodies, only single-chain Fv (scFv) Ab fragments targeting ephrinB2 have shown therapeutic potential, reducing endothelial cell migration and tube-forming in vitro, and angiogenesis and tumor growth in xenografted mice [59]. Interestingly, in this study two anti-ephrinB2 scFvs were found to have similar efficacy, despite only one of them blocking EphB4 binding, suggesting an Eph receptor-independent mechanism. Indeed, as described above, ephrins are themselves capable of signaling. Given the promiscuous nature of Eph-ephrin interactions, where multiple ephrins can bind any given receptor, targeting ligand is likely to be an inefficient way to block receptor activity.

## 6. Co-Targeting and Bispecific Antibodies

Bispecific antibodies (bsAbs) allow binding to two distinct antigens, bringing them into close proximity. Co-targeting of EphA2 and EphA3 has been investigated in GBM, where these receptors are co-expressed in highly tumorigenic stem-like cells [51,65]. Targeting EphA2 and EphA3 co-expressing cells with a cytotoxin linked to the ligand ephrin-A5 initially showed the effectiveness of this approach in GBM models, although not antibody-based [65]. However, subsequent generation of a bsAb against EphA2 and EphA3 showed that even unconjugated antibody was effective in reducing stem cell clonogenicity in vitro, and tumor burden of recurrent GBM xenografts in vivo [51].

Bispecific antibodies recognizing both an Eph receptor and the T cell receptor/CD3 complex on T cells were also generated, in order to recruit T cells to tumors and thereby improve anti-tumor immune responses. The tumor-selective EphA2 antibody EA2 was used in this way to direct unstimulated T cells to lyse EphA2-expressing tumor cells in vitro and in vivo [71]. This approach was also used with an EphA10/CD3 bi-specific antibody, which promoted T cell-mediated tumor cell lysis and inhibited breast cancer xenografts in mice [52].

## 7. Combination Therapies

Since Eph receptors are often expressed on sub-populations of cells within tumors or the TME, anti-Eph antibodies may only target a relatively small proportion of the tumor. Thus, their efficacy will likely be enhanced in combination with established therapies. The choice of combination therapy is generally determined by the approved 1^st^-line therapy used for a given cancer type, and/or use of a drug likely to synergize with the anti-Eph treatment. Established chemotherapies that target the bulk tumor population are an attractive choice for combination with Eph antibodies that target progenitor cells, or cells in the TME, which are less sensitive to chemotherapy [72]. In accordance, the growth inhibition of breast and gastric xenograft models by EphA2 mAb DS-8895a was improved in combination with cisplatin [28]. Likewise, the EphA2 antibody EA5 enhanced effects of the chemotherapy docetaxel in an endometrial cancer murine model [29]. It also enhanced sensitivity to tamoxifen in ER+ breast cancer xenografts, where EphA2 expression is associated with decreased estrogen-dependence [30]. Another more targeted combination therapy explored with anti-EphA2 treatment is the HER2/erbB2 antibody trastuzumab (Herceptin). As mentioned above, cross-talk between EphA2 and the RTK erbB2 was found to promote tumorigenesis and metastasis in mice [15], and in accordance EphA2 expression in human breast cancers was found to correlate with resistance to trastuzumab, and co-treatment with the EphA2 antibody 3F2-3M was shown to restore trastuzumab sensitivity in mice [73].

The other most common combination therapy used in preclinical studies is the anti-angiogenic anti-VEGF antibody bevacizumab, which prevents VEGFR signaling. Since Ephs play a distinct role in neo-angiogenesis, combined inhibition might improve inhibition of tumor vessel formation and tumor growth. Indeed, anti-VEGF treatment was seen to improve therapeutic response to the EphA3 mAb IIIA4 [41], and also enhanced response to antibodies against EphB4 [54], in xenograft models.

## 8. Antibody Payloads

Another approach to take advantage of Ephs as tumor antigens is to use antibodies to carry cytotoxic payloads, in order to selectively kill Eph-expressing tumor cells. Antibody-drug conjugates (ADCs) are now accepted as a therapeutic approach, with four ADCs currently used in the clinic [9]. ADCs use toxins directly conjugated to antibodies using non-cleavable or cleavable linkers, the latter designed to release the active drug upon internalization into target cells (facilitated by proteases, or by reduction or low pH) [74]. The humanized EphA2 mAb 1C1 was conjugated to a microtubule-disrupting auristatin drug via a non-cleavable linker (maleimidocaproyl-MMAF, or mc MMAF). The ADC, generated by MedImmune and known as MEDI-547, effectively killed EphA2+ cells in vitro via caspase-mediated cell death, and more effectively inhibited tumor growth in mouse xenograft models, compared to naked 1C1 [34,35,36].

An ADC directed against EphA3 based on the IIIA4 mAb, and utilizing the microtubule inhibitor maytansine (IIIA4-USAN), was highly effective in killing GBM cells in vitro, and potently inhibited growth of multiple GBM tumor models in mice, where the naked antibody had little effect [48]. Similarly the antagonist EphB2 mAb 2H9 did not inhibit tumor cell proliferation as a naked antibody, but when conjugated to the auristatin MMAE it was effective in inhibiting growth of fibrosarcoma and colon cancer xenografts [53].

An ADC against ephrin A4 was also developed, following identification of the ligand as enriched on tumor-initiating (or stem-like) cells in triple-negative (TN) breast and ovarian patient-derived xenografts [57]. This ADC (PF-06647263) used the humanized mAb E22 linked to the DNA-damaging drug calicheamicin and proved highly effective in suppressing growth of multiple TN breast and ovarian xenograft models [57]. This study also investigated toxicology in monkeys, showing similar organ toxicity to other calicheamicin-based ADCs, suggesting only off-target effects, and the potential for a therapeutic window in cancer patients (see below).

Alternatively, antibodies can be been used to target nanoparticles or liposomes encapsulating cytotoxic drugs to tumors. EphA2 mAb-conjugated liposomes (MM-310) bearing a hydrolytically sensitive docetaxel prodrug have shown promising anti-tumor activity in a range of xenograft models, with improved tumor penetration and anti-tumor activity compared to free docetaxel. Importantly this led to lower levels of free docetaxel in the circulation, and low toxicity in rodents and dogs [40]. Similarly, anti-EphA3 bound nanoparticles loaded with the DNA alkylation agent temozolomide showed tumor targeting, enhanced tumor cell death and increased survival in a rat glioma model [50].

Radioactive isotopes have also been utilized as payloads for Eph antibodies. The EphA2 mAb IF7 coupled to Lutetium-177 showed therapeutic effect in an EphA2-expressing leukemia model with MLL translocation [33]. The therapeutic effect of anti-EphA3 antibody IIIA4 in leukemic models was greatly enhanced by adding an α-particle-emitting ^213^Bismuth payload [47]. In glioblastoma (GBM) models, treatment with IIIA4 labeled with the β-particle-emitting ^177^Lutetium showed dose-dependent tumor cell killing and tumor growth inhibition in vivo, compared to unlabeled antibody [49]. As discussed below, this approach also enables imaging of antibody targeting, for measuring uptake in tumors compared to normal tissues, and for diagnostic or theranostic purposes.

## 9. CAR-T Cells

Another exciting current approach is to use the antigen-binding domains of mAbs to engineer Chimeric Antigen Receptor (CAR)-T cells, to target cytotoxic T cell activity to tumors [10]. CAR-Ts incorporate artificial constructs composed of an extracellular domain harboring the antigen-binding (scFv) fragment of an antibody, linked to transmembrane and cytoplasmic sequences including the T cell receptor CD3 ζ chain, usually with one or more co-stimulatory domains, such as from CD28 (in so-called 2nd and 3rd generation CARs) [10]. EphA2 CAR-Ts based on a humanized version of mAb EA2 [21] (4H5) showed effective tumor cell killing of glioma cells both in vitro and in mouse xenografts, using multiple arrangements of costimulatory signaling domains [75]. Similarly, EphA3-specific CAR-Ts have been developed based on the EphA3 mAb IIIA4/Ifabotuzumab, and demonstrated toxicity against patient-derived GBM cell lines [76]. As with ADCs, further development of these therapeutic approaches will depend on tumor-specific targeting of these agents relative to normal tissues.

## 10. In Vivo Imaging

Antibodies against Eph receptors have been used to image targeting to tumors, as a diagnostic or ‘theranostic’ approach. This is an important step for evaluation of antibody pharmacokinetics and biodistribution in vivo, including tumor uptake, receptor kinetics and saturation, and specificity, preceding clinical testing.

The humanized EphA2 mAb 1C1, labeled with ^64^Cu, was used for positron emission tomography (PET) imaging of eight tumor models with different EphA2 expression levels, showing good correlation between tumor uptake and EphA2 expression. Since the antibody binds both mouse as well as human EphA2, specificity for EphA2 in the tumor compared to normal tissues could also be assessed in mice. This showed uptake in mouse CT-26 colon tumors of around 25% of injected dose/gram (ID/g) at 48 h after injection, with some uptake in liver (around 10%ID/g) and minimal uptake in other tissues, at the same time point [37]. 4B3, a monoclonal antibody specific to human EphA2, was also labeled with ^64^Cu and used for PET/magnetic resonance imaging (MRI) in GBM models, demonstrating clear delineation of tumor boundaries [39].

The anti-EphA2 mAb DS-8895a was radiolabeled for biodistribution, imaging, and pharmacokinetic studies with PET, MRI and SPECT (Single-Photon Emission Computed Tomography). This showed high uptake of ^111^In- and ^89^Zr-labeled mAb in EphA2-expressing xenograft models, with saturation at 30 mg/kg, and no specific uptake in normal tissues [77]. ^89^Zr radiolabeling of the EphA2 mAbs 1C1, 3B10 and 2H7 was also used to compare their uptake as naked mAbs or as ADCs, in tumor cells and in vivo. Interestingly, this showed the hydrophobicity of the mAbs inversely correlated with their uptake, and addition of drug conjugates significantly reduced uptake in vivo [78]. Biodistribution of the EphA3 antibody IIIA4 was also explored, using ^111^In- and ^125^I-labeled antibody, demonstrating clear tumor accumulation in an EphA3-overexpressing xenograft [41].

For EphB4, mAb47 and mAb131 were labeled with ^64^Cu for PET imaging in colon cancer xenografts. This revealed prominent tumor accumulation of mAb47 compared to an IgG control, which, as it binds to both human and mouse EphB4, suggests specificity for tumor over normal tissues. Unsurprisingly, the human-specific mAb131 showed even better accumulation, around 30% ID/g [56]. Near-infrared fluorescence (NIRF) imaging has also been used for these antibodies, by conjugation of the dye Cy5.5 to either full mAbs or F(ab) fragments. mAb131 again showed specific tumor uptake, but the rate of uptake of the F(ab)_2_ was significantly faster, peaking at 6 h compared to 48 h [79].

## 11. Clinical Trials

To date, three anti-Eph antibodies have been tested in clinical trials. The unconjugated anti-EphA2 mAb DS-8895a has been evaluated in a Phase I trial (ClinicalTrials.gov identifier NCT02004717) in Japanese patients with advanced solid tumors [31]. The humanized mAb was afucosylated to enhance ADCC activity, improving antibody binding of the FcγRIIIa receptor (CD16), highly expressed on natural killer (NK) cells. The antibody was well-tolerated, with six dose levels investigated, up to 20 mg/kg, without reaching a maximum tolerated dose. There were two expansion cohorts, EPHA2-positive esophageal and gastric cancer patients. Only 3 of 37 patients had Grade 3 or higher adverse events (AEs) and 51% had manageable infusion reactions, with only 1 patient discontinuing due to drug-related toxicity. One gastric cancer patient showed a partial response and 13 patients achieved stable disease, although the latter did not correlate with EphA2 expression in tumor biopsies, suggesting this may not be treatment-related. Consistent with DS-8895a-induced ADCC activity, a decrease in CD16-positive NK cells and a transient increase in serum inflammatory cytokines were observed after treatment [31]. A second trial to investigate imaging and safety of DS-8895a in non-Japanese patients with advanced EphA2+ tumors (NCT02252211) used more limited treatment doses and incorporated PET imaging of ^89^Zr trace-labeled antibody at day 1 and 36. Encouragingly, specific tumor uptake was observed, and there was no non-specific normal tissue uptake [32]. 

A humanized afucosylated version of the EphA3 antibody IIIA4 (Ifabotuzumab/KB004) was also investigated in a clinical trial (NCT01211691) in patients with EphA3-expressing hematologic malignancies [46]. Treatment was generally well-tolerated, with AEs generally due to transient low-grade infusion reactions. Target plasma concentration exceeding the expected efficacy concentration (of 1 µg/mL) was achieved at weekly doses of 190 mg (approx. 2.6 mg/kg) and above, with the recommended phase 2 dose being 250 mg (approx. 3.5 mg/kg) due to excessive infusion reactions at higher doses. Four responders were observed in the total cohort of 64 patients, in patients with acute myeloid leukemia, myelofibrosis, myelodysplasia and myelodysplasia -myeloproliferation overlap syndrome. Moreover, four patients with AML achieved >50% blast clearance. A subsequent trial assessing KB004 safety and targeting in GBM patients (NCT03374943) is ongoing, with patients treated at 3.5 mg/kg and 5.25 mg/kg doses. Preliminary findings showed rapid, specific accumulation in tumor in all GBM patients, with no normal tissue uptake [45]. MRI images indicated changes to tumor vasculature, consistent with TME targeting of the vasculature. The best response was stable disease for 23 weeks before the patient withdrew from the study for social reasons. This is the first study to demonstrate the feasibility of successfully targeting and imaging EphA3 in the tumor microenvironment, with some early signals of therapeutic effect.

To date, only one ADC against an Eph has been tested in humans. The anti-EphA2 1C1 antibody was tested as an auristatin conjugate, MEDI-547, in a phase I trial (NCT00796055) to investigate safety and maximum tolerated dose in relapsed or refractory solid tumors [38]. Unfortunately, the trial was terminated due to serious adverse events (AEs) including hemorrhage and blood coagulation, likely treatment-related, at the initial dose (0.08 mg/kg). This was despite prior screening of full human tissue panels for binding reactivity, which found generally no staining, apart from some rare and weak staining in some epithelia (e.g., tonsil, esophageal). Non-specific binding had been observed in preclinical models, but at 60-fold higher dose than the clinical starting dose. Minimal dissociation of toxin was detected in the blood, suggesting release of free drug was unlikely to be the cause. The authors concluded that antibody binding to normal tissues required reassessment [38], and it would be important to know whether binding is on-target (specific) or off-target (non-specific). Notwithstanding these results, the overexpression of EphA2 in many tumor types may yet provide a therapeutic window for other antibody-based approaches. Indeed, preclinical studies with the anti-EphA2 immuno-liposome containing docetaxol prodrug (MM-310) did not result in coagulation events in dogs or rats, perhaps due to more restricted biodistribution of the liposomes [40]. As described above, tumor retention was superior to that of the free drug, resulting in effective tumor inhibition. On this evidence, a clinical trial has been initiated (NCT03076372), although no results have yet been posted.

Lastly, the anti-ephrin A4–Calicheamicin ADC (PF-06647263) has been evaluated in a phase I study of 60 patients, the majority with ovarian or breast cancer (NCT02078752) [58]. A dose escalation study of 48 patients started at 0.015 mg/kg, with dose-limiting toxicity above 0.1 mg/kg (3 weekly) due to thrombocytopenia. Dose-expansion was at 0.015 mg/kg (weekly) in TN breast cancer patients. Most patients experienced AEs with around half at grade 3 or 4. Six patients (10%) had a partial response; however, the study was terminated due to limited response to adequate exposure of PF-06647263 in patients [58].

## 12. Conclusions

Aberrant expression and function of Eph receptors in various cancers continues to promote interest in therapeutic targeting of this complex family of receptor tyrosine kinases. Their location on the cell surface in tumors and the supporting microenvironment means they are well suited to targeting by large molecules such as antibodies, which have superior half-life, specificity and affinity, and can initiate multiple tumor-inhibiting responses. Lessons from preclinical and clinical studies highlight the importance of carefully characterizing receptor expression and antibody binding in normal versus tumor cells and tissues using multiple techniques, and fully understanding the effects of treatment response in vivo. However, recent trials showing good toleration, tumor targeting, and some clinical responses, suggest Eph receptor-targeted antibody-based therapies will continue to be a major focus of development.

## Figures and Tables

**Table 1:** Antibodies Against Eph Receptors, Applications, and if Tested in the Clinic.

Target	Antibody	Effects/Application	Clinic	Ref.
EphA2	EA1.2	Agonist, receptor phosphorylation and degradation. Inhibition of tumor cell growth/tube formation	No	[20]
EphA2	EA2, B233	Agonist, tumor cell selective, inhibition of MDA-MB-231 breast tumor growth in vivo	No	[21]
EphA2	EA5	EphA2 degradation in ovarian tumors in vivo, inhibition of tumor growth	No	[22,29,30]
EphA2	IgG25	Agonist, inhibitory in a pancreatic xenograft	No	[24]
EphA2	IgG28	Ligand blocking, inhibitory in a pancreatic xenograft	No	[24]
EphA2	mAb208	Agonist, leading to receptor degradation, promoting CD8 T cell-mediated lysis	No	[25]
EphA2	3F2-3M (B233)	Agonist, induction of ADCC	No	[27]
EphA2	DS-8895a	Antagonist, induction of ADCC; radiolabeled for biodistribution	Yes	[28,31,32]
EphA2	IF7-Lu-177	Radiolabeled mAb inhibited murine leukemia model	No	[33]
EphA2	1C1/MEDI-547	Agonist, receptor phosphorylation and internalization; Auristatin-1C1 ADC (not 1C1 alone) inhibited tumor growth and metastasis in vivo; radiolabeled for biodistribution	Yes	[34,35,36,37,38]
EphA2	4B3	Radio-labeled for PET/MRI biodistribution imaging	No	[39]
EphA2	MM-310	Pro-docetaxol loaded immunoliposomes, tumor targeting and growth inhibition, low organ toxicity	Begun	[40]
EphA3	IIIA4/KB004/ Ifabotuzumab	Agonist, receptor phosphorylation and internalization; Targeted stem-like tumor and TME cells, inhibited growth and vascularity of solid and hematopoietic tumors	Yes	[41,42,43,44,45,46]
EphA3	IIIA4 conjugates	Radioactive or drug payloads inhibited tumor growth in leukemic and GBM models; also in vivo imaging	No	[47,48,49,50]
EphA2/A3	A2/A3 BsAb	Reduced GBM stem-cell qualities and tumor growth in vivo	No	[51]
EphA10	EphA10/CD3 BsAb	Promoted T cell-mediated tumor cell lysis and inhibited breast cancer xenografts	No	[52]
EphB2	2H9	Antagonist. Auristatin-ADC inhibited colon xenograft growth	No	[53,54]
EphB4	C2	Inhibits tumor angiogenesis and growth	No	[55]
EphB4	Mab131, Mab47	Inhibit tumor angiogenesis and growth; radio- labeled to image; 131 induces receptor degradation	No	[54,56]
EphrinA4	PF-06647263	ADC with calicheamicin-γ1, inhibited breast and ovarian xenografts	Yes	[57,58]
EphrinB2	scFv B11, 2B1	Inhibited angiogenesis and pancreatic xenografts	No	[59]
